# Role of Free Radicals in the Pathophysiology of OSA: A Narrative Review of a Double-Edged Sword

**DOI:** 10.3390/jcm14134752

**Published:** 2025-07-04

**Authors:** Alessio Marinelli, Andrea Portacci, Andras Bikov, Pierluigi Carratù, Vitaliano Nicola Quaranta, Zsofia Lazar, Giovanna Elisiana Carpagnano, Silvano Dragonieri

**Affiliations:** 1 Department of Respiratory Diseases, University of Bari, 70121 Bari, Italy; alessio.marinelli@uniba.it (A.M.); a.portacci01@gmail.com (A.P.); vitalianonicola.40@gmail.com (V.N.Q.); elisiana.carpagnano@uniba.it (G.E.C.); silvano.dragonieri@uniba.it (S.D.); 2 Wythenshawe Hospital, Manchester University NHS Foundation Trust, Manchester Academic Health Science Centre, Manchester M23 9WL, UK; andras.bikov@gmail.com; 3 Division of Infection, Immunity and Respiratory Medicine, Faculty of Biology, Medicine and Health, The University of Manchester, Manchester M13 9PL, UK; 4 Internal Medicine “A. Murri”, University of Bari, 70121 Bari, Italy; 5 Department of Pulmonology, Semmelweis University, 1085 Budapest, Hungary; lazar.zsofia@semmelweis.hu

**Keywords:** OSAS, oxidative, ROS, CPAP, antioxidant

## Abstract

Obstructive sleep apnea (OSA) is a highly prevalent sleep-related breathing disorder, primarily characterized by recurrent episodes of upper airway obstruction during sleep. Individuals affected by OSA are at increased risk for a variety of adverse health outcomes, particularly neurocognitive impairments and cardiovascular complications, highlighting the clinical significance of this condition. A defining feature of OSA is intermittent hypoxemia, which contributes to the excessive production of reactive oxygen species (ROS) and the subsequent development of oxidative stress. The primary objective of this narrative review was to comprehensively investigate the intricate mechanisms of oxidative stress and elucidate their complex interplay in the development and progression of OSAS. Subsequently, we examined the current literature to identify the most promising biomarkers and pharmacological treatments related to OSA and oxidative stress. We found that biomarkers of oxidative stress have shown potential in assessing disease severity and tracking individual responses to therapy. However, none have yet to be incorporated into standard clinical practice. Continuous positive airway pressure (CPAP) is the gold standard treatment for OSA. Nevertheless, antioxidant therapy has emerged as a potential adjunctive approach that may help address residual dysfunctions not fully resolved by CPAP alone. Both the use of oxidative stress biomarkers and antioxidant-based therapies require further validation through robust clinical studies before they can be routinely implemented in clinical settings.

## 1. Introduction

Obstructive sleep apnea (OSA) represents a prevalent sleep-related breathing disorder (SDB), fundamentally characterized by recurrent episodes of upper airway collapse during sleep. This physiological disturbance leads to a reduction in inspiratory airflow, manifesting either as a complete cessation (apnea) or a partial diminution (hypopnea). 

The estimated prevalence of OSA exhibits considerable variability within the general population, depending on a multitude of factors. These include the specific attributes of the studied cohort (e.g., sex ratio, BMI, ethnicity), the methodologies employed for the assessment of SDB, the operational definitions of the disease state (e.g., the criteria for hypopnea), and the threshold of the Apnea/Hypopnea Index (AHI) utilized for OSA diagnosis.

A comprehensive investigation conducted by Benjafield and colleagues in 2019 [1] projected that approximately one billion adults aged between 30 and 69 years globally may be afflicted by obstructive sleep apnea. Furthermore, their analysis indicated that the number of individuals experiencing moderate to severe obstructive sleep apnea, a condition for which therapeutic intervention is generally indicated, approaches 425 million. 

Obstructive sleep apnea can manifest with or without overt clinical symptomatology. Irrespective of symptomatic presentation, individuals diagnosed with OSA exhibit an elevated susceptibility to a range of adverse clinical outcomes, especially neurocognitive and cardiovascular sequelae, underscoring its clinical importance. In line with this, OSA is associated with a substantial economic and societal burden [2,3].

### 1.1. Cardiovascular and Cerebrovascular Morbidity

Patients with OSA, particularly those with moderate or severe disease, face an increased risk of developing systemic hypertension, coronary artery disease, cardiac arrhythmias, heart failure, and cerebrovascular events such as stroke [4].

The recurrent episodes of upper airway obstruction during sleep are associated with intermittent hypoxemia, potential hypercapnia, alterations in intrathoracic pressure dynamics, and recurrent microarousals. The resulting hemodynamic, autonomic, and metabolic perturbations, coupled with systemic inflammation and oxidative stress, are implicated in the pathogenesis of cardiovascular diseases in the context of OSA [5].

OSA is consistently linked to a significant augmentation of sympathetic nervous system activity during sleep. This heightened sympathetic tone contributes to the attenuation of the normal nocturnal decline in blood pressure and heart rate, ultimately predisposing individuals to systemic hypertension [6]. The increased sympathetic activity appears to be mediated through a complex interplay of mechanisms, including chemoreflex stimulation triggered by hypoxemia and hypercapnia, baroreflex modulation, pulmonary afferent signaling, impaired venous return to the heart, alterations in cardiac output, and potentially an arousal. Dyslipidaemia [7], endothelial dysfunction [8], and hypercoagulation [9] may also play a significant role in this process.

OSA has been independently associated with an elevated risk of ischemic stroke, even after accounting for traditional vascular risk factors [10,11]. Several potential mechanisms may underlie this increased stroke risk in patients with OSA. One plausible explanation involves the reduction in cerebral blood flow velocity resulting from the negative intrathoracic pressure typically generated during an obstructive respiratory event. Alternatively, the cerebrovascular dilatory responses to hypoxia may be blunted in individuals with OSA due to the effects of intermittent hypoxia, oxidant-mediated endothelial dysfunction, increased sympathetic activity, and impaired cerebral vasomotor reactivity to carbon dioxide [12]. These recurrent reductions in cerebral blood flow velocity can then precipitate ischemic changes, particularly in vulnerable border-zone areas and terminal arterial territories, especially in patients with a compromised hemodynamic reserve (e.g., those with intracranial arterial stenosis) [13]. Furthermore, OSA may exacerbate pre-existing cerebrovascular abnormalities or other established risk factors for stroke [10]. Supporting this notion, patients with OSA exhibit a higher prevalence of systemic hypertension, heart disease, impaired vascular endothelial function, accelerated atherogenesis, diabetes mellitus, atrial fibrillation, prothrombotic coagulation shifts, proinflammatory states, and increased platelet aggregation [14].

Pulmonary hypertension and right heart failure are also recognized complications of OSA. Classically, OSA is associated with group 3 pulmonary hypertension, particularly when OSA coexists with obesity hypoventilation syndrome or other conditions causing daytime hypoxemia, such as chronic lung disease [15,16].

### 1.2. Neuropsychiatric Dysfunction

OSA can induce or exacerbate deficits in attention, memory, and overall cognitive function. These impairments can collectively lead to compromised executive function, consequently elevating the propensity for errors and accidents [2,17]. Notably, the incidence of motor vehicle accidents is two to three times higher among individuals with OSA compared to those without the disorder [18]. Additional neuropsychiatric manifestations associated with OSA include increased mood lability and irritability, as well as a higher prevalence of depression, psychosis, and sexual dysfunction [19,20].

### 1.3. Metabolic Syndrome and Type 2 Diabetes

Individuals with OSA demonstrate an increased prevalence of insulin resistance, as well as type 2 diabetes mellitus and its associated complications [21]. While this association may be partially explained by shared risk factors such as obesity, numerous studies have reported an independent correlation between OSA severity, insulin resistance, and the development of type 2 diabetes [22,23,24]. In patients with the metabolic syndrome, OSA has been independently linked to elevated glucose and triglyceride levels, as well as increased markers of inflammation, arterial stiffness, and atherosclerosis. These findings suggest that OSA may exacerbate the cardiometabolic risks attributed to obesity and the metabolic syndrome [25].

### 1.4. Nonalcoholic Fatty Liver Disease (NAFLD)

Patients with OSA, particularly those with severe OSA characterized by nocturnal hypoxemia and endothelial dysfunction, exhibit a two- to three-fold increased prevalence of NAFLD. This association appears to be independent of shared risk factors such as obesity [26].

### 1.5. Miscellaneous

Emerging evidence suggests that individuals with OSA may have a higher risk of developing gout compared to those without OSA [27]. Furthermore, a large retrospective study based on a French cohort indicated a potential association between cancer and nocturnal hypoxemia in patients undergoing investigation for OSA [28]. Another study proposed a slight increase in the risk of unprovoked venous thromboembolism in OSA patients experiencing severe nocturnal hypoxemia [29].

Individuals diagnosed with OSA, especially those with moderate to severe manifestations of the condition who do not receive therapeutic intervention, demonstrate a markedly increased vulnerability to a spectrum of detrimental clinical sequelae. As highlighted by accumulating evidence, the heightened oxidative stress resulting from the characteristic intermittent hypoxia in OSA is increasingly recognized as a pivotal factor in the pathogenesis of the various comorbidities observed in this disorder. Consequently, the primary objective of this narrative review was to comprehensively investigate the intricate mechanisms of oxidative stress and elucidate their complex interplay in the development and progression of OSA.

## 2. Materials and Methods

**Figure 1:**
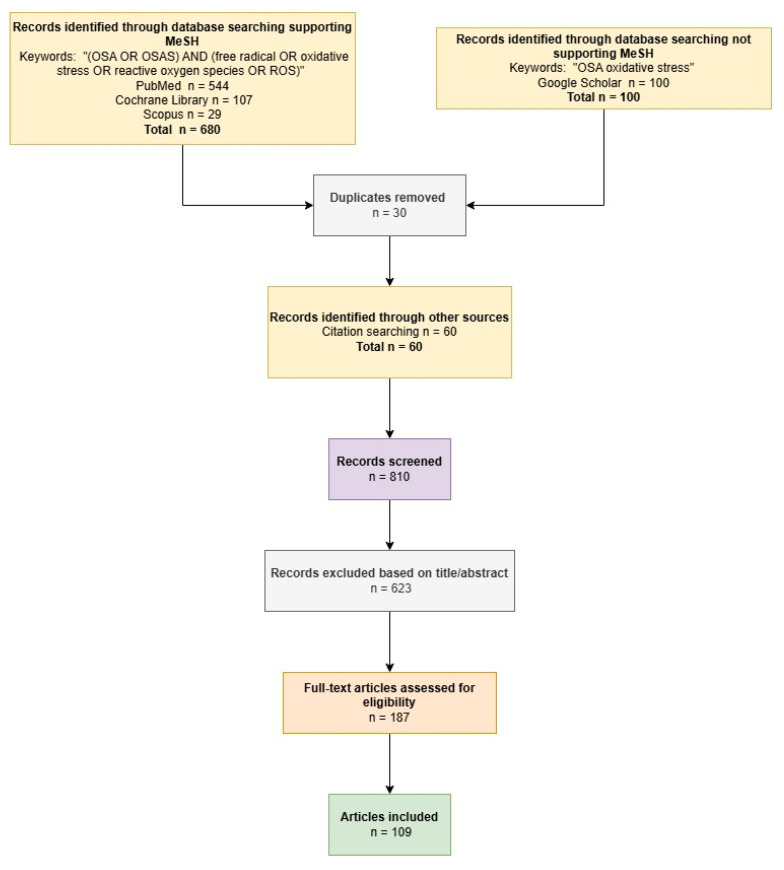
Flowchart outlining search strategy and selection of articles.

A comprehensive search of the literature was executed across several prominent electronic databases, namely PubMed (National Library of Medicine, Bethesda, MD, USA), Cochrane Library (John Wiley & Sons, Inc., Hoboken, NJ, USA), Scopus (The Elsevier Foundation, Amsterdam, NX, Netherlands), and Google Scholar (Alphabet Inc., Mountain View, CA, USA), to identify pertinent studies for this narrative review (see Figure 1).

For the databases PubMed/Medline, Cochrane Library, and Scopus a targeted search strategy was employed utilizing Medical Subject Headings (MeSH) terms and Boolean operators. The specific search syntax implemented was “(OSA OR OSAS) AND (free radical OR oxidative stress OR reactive oxygen species OR ROS)”. To ensure the relevance and accessibility of the retrieved literature, the search was constrained to articles published in the English language for which full-text versions were available. The initial phase of article selection involved a thorough examination of titles and abstracts to identify potentially relevant publications. Furthermore, the reference lists of identified articles of interest were scrutinized to uncover additional pertinent literature that may not have been captured by the primary database searches. Two independent researchers (AM and SD) screened the titles and abstracts of all retrieved articles, with a particular emphasis on those published in journals ranked within the first quartile (Q1) of their respective subject categories. Any discrepancies arising during this initial screening process were resolved through collegial discussion and mutual agreement on the articles warranting a full-text review.

For the Google Scholar database, a more streamlined search syntax, “OSA oxidative stress”, was utilized. Similarly to the other databases, the search was limited to the first 100 articles available in English with full-text access. To optimize the efficiency of the initial screening process, the results were prioritized based on citation count, with studies exhibiting the highest citation scores being reviewed first. The same two researchers (AM and SD) independently evaluated the titles and abstracts of the articles retrieved from Google Scholar. Any disagreements encountered during this stage were resolved through collaborative discussion to determine which articles would proceed to a full-text assessment. 

Following the initial retrieval and screening phases, duplicate records were removed to ensure the uniqueness of the identified literature. Subsequently, the full-text versions of the remaining articles were independently assessed for eligibility by the two researchers. Once again, any disagreements regarding the inclusion or exclusion of specific articles were resolved through comprehensive discussion and the establishment of a consensus.

It is important to explicitly state that this narrative review is predicated upon the synthesis of previously conducted studies and does not encompass any original research involving animal subjects undertaken by the authors of this manuscript. It should be noted, however, that some of the studies cited herein include analyses or investigations involving human participants, which were conducted and completed prior to the commencement of the present work.

### Limitations

We acknowledge several limitations in this review article. Primarily, the narrative design inherently lacks systematic rigor. Furthermore, our research methods were constrained to articles published in the English language, and the availability of full-text versions for all retrieved articles may introduce bias.

Another potential limitation is our screening process, which focused on the titles and abstracts of articles published exclusively in journals ranked within the first quartile (Q1) of their respective subject categories. While this selective approach might be perceived as a limitation, we also believe it lends consistency and strength to the evidence presented.

Similarly, our initial search on Google Scholar, which prioritized studies with the highest citation scores, may introduce a similar bias to that of limiting our search to Q1 journals, potentially narrowing the scope of our findings. Further, by limiting our initial screening to the first 100 records on Google Scholar due to the large volume of results (over 18,000), we may have missed relevant studies that were not ranked highly by the database’s algorithm.

## 3. OSA and Intermittent Hypoxia: The Main Driver of Oxidative Stress

**Figure 2:**
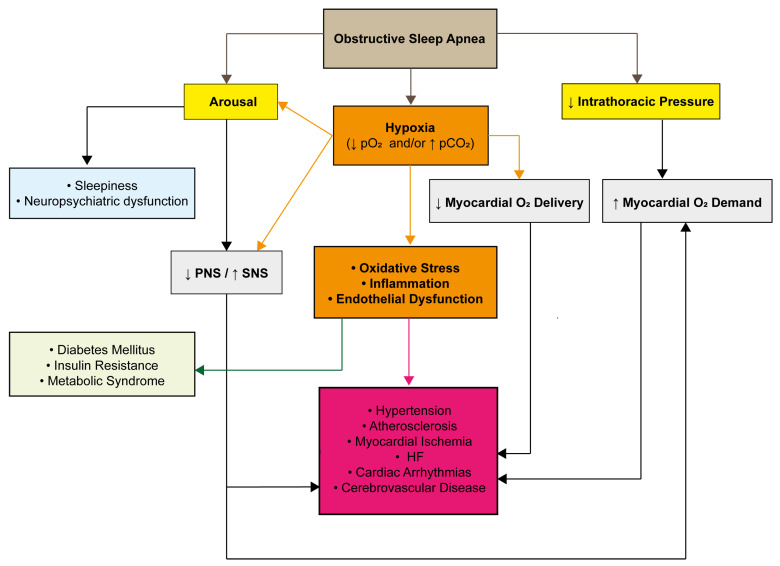
Pathophysiology of obstructive sleep apnea. PNS: parasympathetic nervous system; SNS: sympathetic nervous system; and HF: heart failure.

OSA is marked by repeated episodes during sleep where the upper airway becomes partially or completely blocked. This mechanical obstruction disrupts normal breathing and sets off a chain of physiological responses throughout the body (Figure 2). These episodes often involve reduced or halted airflow despite the continued effort to breathe, leading to repeated drops in oxygen levels (intermittent hypoxemia) and frequent sleep disruptions (arousal) [4,30]. Over time, these disturbances can lead to a wide range of systemic health issues.

During an apnea episode, the body attempts to inhale against a blocked airway, creating strong negative pressure inside the chest. These pressure swings can affect cardiovascular function by increasing the release of atrial natriuretic peptide (ANP), raising left ventricular transmural pressure, and impairing the heart’s ability to fill properly. This ultimately puts more strain on the heart by increasing both preload and afterload. At the same time, the repeated oxygen drops cause an imbalance between the heart’s oxygen needs and the amount actually delivered, which is further worsened by a limited blood flow. In response to low oxygen, high carbon dioxide, and frequent arousals from sleep, the sympathetic nervous system becomes overactive, tightening blood vessels and increasing both heart rate and blood pressure.

**Figure 3:**
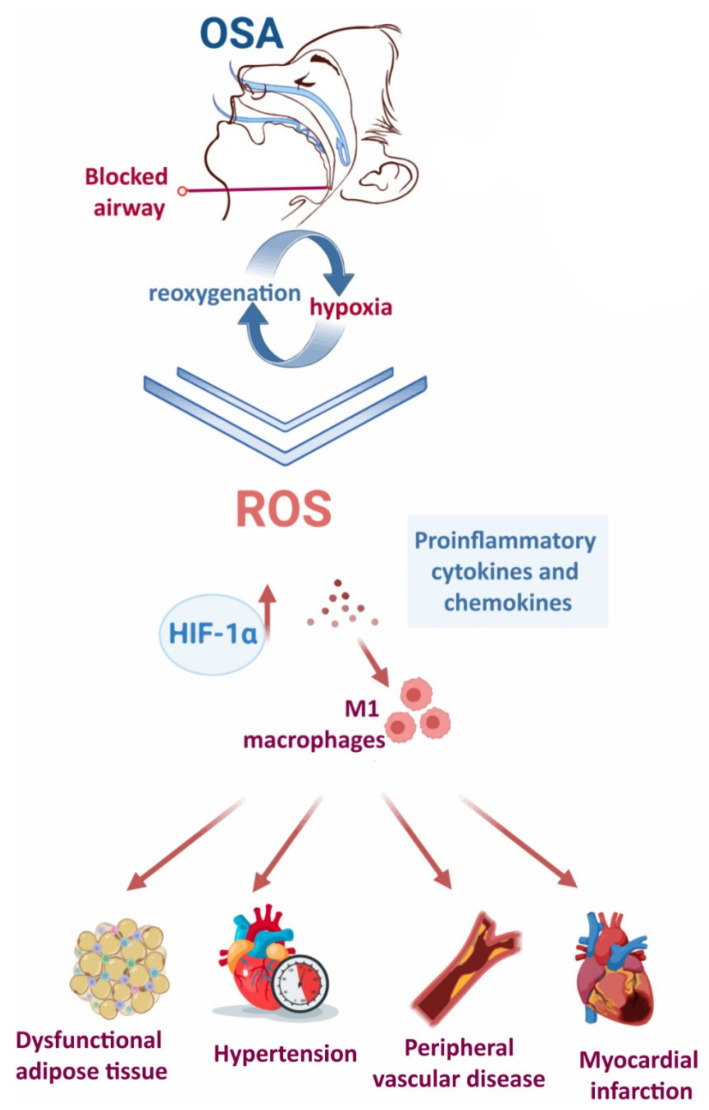
The repetitive cycles of hypoxia–reoxygenation due to frequent blockage of the upper airway in people with OSA triggers the activation of various pathological mechanisms which drives the overproduction of ROS, leading to systemic damage. Image modified from Alterki et al. [31].

One of the hallmark features of OSA is the repeated pattern of intermittent hypoxemia, which drives the overproduction of reactive oxygen species (ROS), leading to oxidative stress [31] (Figure 3).

This intermittent drop-and-rebound pattern in oxygen levels is strikingly similar to what happens in ischemia–reperfusion (I/R) injury, where tissue damage is caused not just by the lack of oxygen but also by its sudden return. In OSA, each breathing pause followed by reoxygenation mimics this cycle, promoting ROS production through similar mechanisms [32,33].

ROS are unstable molecules that can damage key cellular components like DNA, proteins, and lipids. This can lead to inflammation and broader tissue injury [34]. Additionally, the low-oxygen environment in OSA triggers the release of proinflammatory substances, creating a chronic, low-level inflammatory state. This further disrupts metabolism and encourages platelet clumping, both of which raise the risk for cardiovascular problems [35].

Together, oxidative stress and inflammation are now seen as central to the complex disease process of OSA [36]. Oxidative stress arises when the production of free radicals like ROS surpasses the body’s antioxidant defenses. Inflammation, in turn, is a biological response to these and other stressors—one that can itself be triggered by oxidative stress, creating a feedback loop that fuels ongoing damage.

Apnea events are usually terminated by a microarousal due to growing chemical and mechanical cues from the body, such as falling oxygen levels, rising CO_2_, and the increased effort to breathe. While these arousals are necessary to resume breathing, they also disrupt the normal sleep cycle, contributing to sleep fragmentation and adding to the overall burden of the disease.

## 4. Molecular Mechanisms of Free Radical Generation in OSA

Reactive oxygen species (ROS) are chemically reactive molecules derived from oxygen metabolism and are routinely produced within biological systems. Although ROS are widely recognized for their potential to cause cellular damage—affecting lipids, proteins, nucleic acids, and other macromolecules—often referred to as their “bad” or “ugly” effects, they also serve crucial functions in the regulation of numerous physiological processes, representing their “good” aspects [32,37,38] (Table 1). This dual nature of ROS, often likened to a double-edged sword, underscores the importance of maintaining a finely tuned balance between their production and elimination.

Superoxide anion (O_2_^−^) is commonly the initial ROS formed during aerobic metabolism and can be further converted into hydrogen peroxide (H_2_O_2_) and, subsequently, into hydroxyl radicals (OH^−^), which are among the most reactive and cytotoxic ROS [32]. These conversions are often facilitated by the presence of transition metals, particularly iron (Fe_2_^+^) and copper (Cu^+^), via Fenton and Haber–Weiss reactions. In immune cells such as neutrophils, hypochlorous acid (HOCl) is generated through the activity of myeloperoxidase, contributing to microbial killing. Moreover, the reaction of O_2_^−^ with nitric oxide (NO)–produced by nitric oxide synthase (NOS)–yields peroxynitrite (ONOO^−^), a potent oxidant capable of inducing significant biomolecular damage [39].

While superoxide is generally the most abundant ROS, hydroxyl radicals are considered the most reactive and destructive. Lipid membranes are particularly susceptible to ROS-mediated damage, especially via hydroxyl radical-induced lipid peroxidation, which can initiate chain reactions that compromise membrane integrity [40,41]. DNA is also a critical target; ROS can induce base modifications, strand breaks, and sugar backbone cleavage. The formation of 8-hydroxyguanine (8-OH-G) is frequently used as a biomarker of oxidative DNA damage [42]. Similarly, proteins and small-molecule antioxidants such as glutathione (GSH) are vulnerable to ROS, leading to impaired cellular redox buffering, mitochondrial dysfunction, and diminished contractile and neuronal function [39].

Endogenously, mitochondria and NADPH oxidases (NOX) represent the principal sources of ROS. In mitochondria, ROS arise as by-products of electron leakage from the electron transport chain during oxidative phosphorylation. NOX enzymes, localized in various cellular membranes including those of the sarcolemma, sarcoplasmic reticulum, and transverse tubules, also contribute significantly to ROS generation. Other intracellular sources include xanthine oxidase, uncoupled NOS, and organelles such as peroxisomes and the endoplasmic reticulum. Additionally, numerous immune and vascular cell types—including macrophages, endothelial cells, and polymorphonuclear leukocytes—are known to produce ROS, especially under stress conditions. Exogenous factors such as tobacco smoke, environmental toxins, ionizing radiation, and hypoxic environments further exacerbate ROS levels. 

To preserve cellular integrity and function, cells possess an array of antioxidant defense systems that maintain redox homeostasis. These include enzymatic antioxidants such as superoxide dismutases (SODs), catalase, glutathione peroxidase (GPx), and heme oxygenase-1, as well as non-enzymatic antioxidants like vitamin C, vitamin E, carotenoids, polyphenols, and glutathione. Collectively, these systems modulate oxidative stress and regulate redox-sensitive signaling pathways essential for processes like proliferation, differentiation, and apoptosis. ROS also influence the activity of several transcription factors, including hypoxia-inducible factor-1*α* (HIF-1*α*), nuclear factor *κ*B (NF-*κ*B), activator protein-1 (AP-1), and nuclear factor erythroid 2-related factor 2 (Nrf2), all of which play roles in stress responses and inflammation [43,44].

Among antioxidant enzymes, SOD is especially critical for neutralizing superoxide by catalyzing its conversion into H_2_O_2_ and molecular oxygen. There are three major isoforms of SOD: the cytosolic Cu/Zn-SOD (SOD1), mitochondrial Mn-SOD (SOD2), and extracellular SOD (SOD3). SOD2, in particular, plays a key role in protecting mitochondrial integrity during periods of oxidative stress or ischemia–reperfusion injury [45]. Hydrogen peroxide generated by SOD is further detoxified into water by catalase or GPx, preventing the formation of more reactive species such as OH^−^.

Glutathione (GSH), a tripeptide composed of glutamate, cysteine, and glycine, represents the most abundant intracellular non-enzymatic antioxidant. It plays diverse roles in redox homeostasis, including scavenging ROS, detoxifying hydrogen peroxide and lipid peroxides, and maintaining protein thiol groups in their reduced states. During these processes, GSH is oxidized to glutathione disulfide (GSSG), which can be recycled back to GSH by glutathione reductase. Within the nucleus, GSH helps regulate DNA repair enzymes by maintaining their functional redox state. The cellular GSH/GSSG ratio is widely accepted as an indicator of oxidative stress and redox status [44].

In summary, while ROS are often viewed through the lens of cellular injury, they are also indispensable signaling molecules under physiological conditions. Their effects are highly context-dependent, varying across cell types, tissue environments, and the severity of oxidative stimuli. In conditions like OSA, the interplay between ROS production and antioxidant defense is particularly intricate, with evidence suggesting that the compensatory upregulation of antioxidant pathways occurs alongside elevated oxidative stress. The net outcome hinges on the dynamic balance between prooxidant and antioxidant forces, ultimately influencing disease progression and therapeutic response.

## 5. Oxidative Stress Biomarkers in OSA

Oxidative stress and the subsequent oxidant-induced damage are commonly induced by pathological conditions such as ischemia–reperfusion (I/R) injury and inflammation, both of which disrupt the delicate equilibrium of the redox system. Intermittent hypoxia (IH), a hallmark of OSA, activates various cellular components—including leukocytes, platelets, and endothelial cells—shifting them towards a proinflammatory state. This activation enhances the production of ROS, inflammatory cytokines, and adhesion molecules [32]. These interconnected molecular events culminate in endothelial damage, contributing to oxidative stress and endothelial dysfunction [46]. 

Biomarkers indicative of oxidative stress and antioxidant imbalance have been detected primarily in the blood and other body fluids, such as urine and saliva.

Elevated levels of NF-*κ*B have been linked to OSA due to intermittent hypoxia [36]: NF-*κ*B represents a family of inducible transcription factors, which regulate a large array of genes involved in different processes of the immune and inflammatory responses. As a result, cytokines—such as IL-1, IL-6, IL-12, TNF-*α*–, and chemokines—involved in various inflammatory processes are consequently increased [47]. Therefore, high-sensitivity C-Reactive Protein (hsCRP), traditionally an acute-phase reactant and inflammatory marker, has also emerged as a potential oxidative stress biomarker. Elevated hsCRP levels have been positively correlated with OSA severity parameters, such as the apnea–hypopnea index (AHI), oxygen desaturation index (ODI), and the percentage of total sleep time with oxygen saturation below 90% (TSpO_2_ < 90%) [35,48,49,50].

Biological membranes are particularly vulnerable to damage from free radicals because the unsaturated lipids within them are highly susceptible to oxidation. This process, known as lipid peroxidation, involves the reaction of polyunsaturated fatty acids (PUFAs) present in cellular membrane phospholipids with oxygen, leading to the formation of lipid hydroperoxides. Lipid peroxidation has been a subject of extensive study in OSA, with plasma and serum markers being the most commonly analyzed indicators. Markers have also been detected in exhaled breath condensates [51,52]. Among the primary end products of lipid peroxidation are compounds such as isoprostanes, isofurans, and malondialdehyde (MDA), each arising from distinct oxidative processes involving PUFAs. Isoprostanes and isofurans result from the non-enzymatic oxidation of arachidonic acid (AA) and docosahex-aenoic acid (DHA), respectively. Notably, F2-isoprostanes are prostaglandin-like molecules that emerge through the free radical-induced peroxidation of AA within phospholipids, occurring both in vivo and in vitro. Unlike classical prostaglandins, their formation does not require cyclooxygenase activity. Initially integrated within membrane phospholipids, these compounds are subsequently liberated into circulation. Compared to other lipid peroxidation products, F2-isoprostanes exhibit relatively greater stability and lower reactivity [53] and their levels in biological fluids have been correlated to oxidative stress under different pathophysiologic conditions, including OSA [52,54].

Malondialdehyde (MDA), a well-characterized low-molecular-weight aldehyde, is another significant lipid peroxidation by-product. Due to its high reactivity, MDA readily forms adducts with nucleic acids and proteins, contributing to cellular toxicity. The thiobarbituric acid reactive substances (TBARS) assay is widely employed to quantify MDA levels, although it also detects other lipid peroxides. TBARS concentrations are commonly used as indicators of oxidative lipid damage and have shown a positive correlation with clinical measures of OSA severity, such AHI and ODI [53,55].

As plasma proteins are one the first targets of free radicals, the detection of Advanced Oxidation Protein Products (AOPPs) in biologic fluids can be an optimal strategy, which represent oxidatively modified proteins, and serve as indicators of both oxidative stress and inflammatory processes [53,56]. AOPPs are considered more stable than lipid oxidation markers [57]. There is a significant correlation between circulating AOPP levels and markers of disease severity such as AHI, ODI, and TSpO_2_ < 90%. Moreover, patients with moderate to severe OSA displayed significantly higher AOPP concentrations compared to those with mild or no disease [58,59,60].

Another oxidative stress biomarker, 8-hydroxy-2-deoxyguanosine (8-OHdG), reflects oxidative damage to DNA. Increased urinary excretion of 8-OHdG has been observed in individuals with severe OSA, with positive associations reported between 8-OHdG levels and AHI, ODI, and TSpO_2_ < 90% [61].

Superoxide dismutase (SOD), a crucial antioxidant enzyme that catalyzes the dismutation of superoxide radicals, shows diminished activity in patients with OSA compared to healthy controls, indicating compromised antioxidant defense [62]. A potential mechanism for lower SOD levels in OSA could be the reduced production of the klotho protein [63] that is a known inducer of SOD [64].

Similarly, the thioredoxin (Trx) system—comprising Trx, NADPH, and thioredoxin reductase (TrxR)—is involved in maintaining redox homeostasis and regulating gene expression. Trx concentrations have been positively associated with OSA severity, as reflected by increased AHI and decreased oxygen saturation levels [65,66].

Heme oxygenase is primarily responsible for heme degradation. It is induced by ROS and leads to the formation of carbon monoxide (CO), ferrous iron, and biliverdin. The latter has antioxidant and anti-inflammatory properties [67]. Exhaled CO, which reflects on the heme oxygenase activity and the corresponding oxidative stress in the airways, has shown to be elevated in OSA [68].

Although numerous genetic polymorphisms have been implicated in OSA pathogenesis, none have achieved validation as diagnostic or screening tools in clinical settings. Likewise, while microRNAs hold diagnostic potential, they have not yet been integrated into clinical practice due to the absence of robust validation studies [36].

At present, the most robust biomarkers of oxidative stress in OSA include hsCRP, thioredoxin, TBARS, AOPPs, and 8-OHdG (see Table 2 and Table 3). Despite numerous efforts to identify reliable markers for diagnosis and disease severity, the current body of evidence has not yielded consistent or clinically applicable results [35,36,50,69].

## 6. Discussion

More than two decades ago, the involvement of ROS in the pathophysiology of OSA was largely hypothetical. Early theories proposed that recurrent hypoxia during sleep leads to elevated levels of free radicals, which in turn contribute to inflammation and the development of atherosclerosis [74]. While considerable progress has since been made in understanding the biological implications of oxidative stress in OSA, continuous positive airway pressure (CPAP) therapy remains the only well-established intervention with proven antioxidant effects. By restoring normal nocturnal oxygen saturation, CPAP disrupts the molecular pathways leading to oxidative damage, thereby modulating the expression of various proinflammatory and pro-thrombotic mediators [52,75,76,77,78].

While CPAP remains the cornerstone of therapy for OSA, suboptimal adherence has prompted significant investigation into alternative treatments. A summary of current evidence for these alternatives is presented below.

### 6.1. Oral Appliances

Intraoral devices, particularly Mandibular Advancement Devices (MADs), represent a significant alternative to CPAP. These appliances function by repositioning the mandible to increase airway caliber and reduce collapsibility during sleep [79]. A joint systematic review from the American Academy of Sleep Medicine and the American Academy of Dental Sleep Medicine confirmed that custom-fitted oral appliances reduce the frequency of respiratory events (e.g., AHI, RDI) and offer a modest improvement in minimal oxygen saturation. While they do not significantly alter sleep architecture, they have been shown to improve daytime sleepiness and quality of life to a degree that is nearly comparable to CPAP [80]. Similarly, their modest blood pressure-lowering effects are considered almost equivalent to those of CPAP therapy [81].

Crucially, patient adherence to oral appliances is frequently higher than to CPAP therapy [82], and the recent integration of temperature microsensors allows for objective adherence monitoring. Consequently, oral appliances are a well-founded option for individuals with primary snoring or mild to moderate OSA, especially those who cannot tolerate or fail CPAP. Patient selection can be refined by identifying specific phenotypic traits associated with a favorable response, such as female sex, lower BMI, and certain craniofacial characteristics [83]. Nonetheless, for severe OSA, CPAP should be trialed first. Follow-up sleep testing is recommended to confirm efficacy, as the overall success rate for reducing the AHI to below 10 events/h is estimated at approximately 50% [84].

### 6.2. Positional Therapy

It is well-established that sleeping posture affects airway patency, with the supine position increasing the likelihood of obstruction due to gravitational effects on the tongue and greater airway collapsibility [85,86]. For patients with position-dependent OSA, therapy aimed at maintaining a lateral decubitus position can be effective, such as the use of a bulky item, such as a tennis ball, placed on the patient’s back [87]. While some studies suggest equivalence to CPAP in this specific patient subset [88], a Cochrane review found positional therapy to be inferior in reducing the AHI and improving oxygenation. Long-term adherence, while suboptimal, appears better than for CPAP, but without superior outcomes in quality of life [89]. Combining positional therapy with a MAD may yield synergistic effects and a greater AHI reduction [90].

### 6.3. Myofunctional Therapy

The function of the upper airway musculature is a key factor in maintaining pharyngeal patency. Accordingly, researchers have investigated targeted exercises and other training modalities as a potential therapy for OSA. 

Myofunctional therapy (MT) is composed of isotonic and isometric exercises that target oral (lip, tongue) and oropharyngeal structures (soft palate, lateral pharyngeal wall) [91].

A systematic review and meta-analysis by Camacho et al. [92] evaluated MT as a treatment for OSA in children and adults. The analysis showed that MT decreases the AHI by approximately 50% in adults and 62% in children. Furthermore, the authors reported a 72% reduction in snoring and a decrease in the Epworth Sleepiness Scale (ESS) score from 14.8 ± 3.5 to 8.2 ± 4.1. While these results are promising, the meta-analysis has several limitations. It included only eleven studies: nine involving adults and two involving pediatric patients. Moreover, only one study reported outcomes for longer than six months after the initiation of MT exercises; the follow-up periods for all other studies spanned from two to six months. Therefore, additional long-term studies are needed to demonstrate the lasting effects of continued MT.

### 6.4. Surgical Interventions

A variety of surgical procedures are available, though their efficacy, with the notable exception of tracheostomy, is generally considered less than that of CPAP [93].

- Hypoglossal Nerve Stimulation (HGNS): This therapy involves stimulating the hypoglossal nerve to induce genioglossus muscle contraction, thereby stabilizing the airway. Systematic reviews have confirmed its efficacy in reducing AHI and improving sleepiness and quality of life in appropriately selected patients with moderate to severe OSA [94].
- Tracheostomy: This procedure is nearly 100% effective at resolving obstructive events [95] but is reserved for patients with the most severe OSA who have failed all other therapies and exhibit life-threatening complications [96].
- Maxillomandibular Advancement (MMA): This invasive surgery expands the facial skeleton to permanently enlarge the airway. For carefully selected patients with a refractory to other treatments, it is highly effective, with one meta-analysis reporting a mean AHI reduction of over 80% [97].
- Uvulopalatopharyngoplasty (UPPP): The long-term efficacy of UPPP is questionable. While an initial reduction in AHI may be observed, this effect tends to diminish significantly over time [98]. It is not recommended as a standalone procedure due to its inconsistent outcomes [99].

### 6.5. Oxygen Therapy

The role of supplemental oxygen in OSA management is limited. While it effectively corrects nocturnal desaturation, it does not treat the underlying obstruction and may even prolong apneic events by increasing the arousal threshold [100]. In certain patients with high loop gain, it can stabilize ventilatory control and reduce the AHI [101], but it is not considered a definitive treatment for OSA [102].

### 6.6. Pharmacologic Therapy

An emerging area of research is pharmacotherapy. A combination of atomoxetine and oxybutynin has shown promise by enhancing genioglossus muscle activity, leading to a significant reduction in AHI. This effect was not observed when either drug was administered alone, suggesting a synergistic mechanism. However, more extensive investigation is required before this can be considered for widespread clinical use [103]. 

To date, only a limited number of studies have evaluated antioxidant agents as therapeutic options aimed at mitigating oxidative stress in OSA patients (see Table 4), and their clinical efficacy remains uncertain. 

Sadasivam et al. [104] evaluated the effects of N-acetylcysteine (NAC) as an antioxidant therapy in individuals with OSA. Twenty patients with OSAS were enrolled in the study over a 30-day period. After polysomnography (PSG), they were randomly assigned to receive a placebo (n = 10) and NAC (n = 10). To assess oxidative stress and antioxidant response, the study measured lipid peroxidation levels, which showed a statistically significant reduction in the NAC-treated group compared to the control group (*p* < 0.001). Concurrently, glutathione concentrations increased significantly in treated individuals but remained unchanged in controls (*p* < 0.001). Beyond biochemical outcomes, improvements were also noted in sleep-related parameters, including sleep architecture, efficiency, respiratory function, and snoring. NAC serves as a precursor for glutathione synthesis and has demonstrated superior antioxidant properties compared to other agents, not only by neutralizing reactive oxygen species such as superoxide radicals, but also by enhancing endogenous glutathione production—a key intracellular antioxidant. Additionally, NAC is frequently utilized in clinical settings to manage conditions marked by systemic inflammation. While these findings indicate that NAC may reduce oxidative stress, the study’s small sample size and short duration of administration are insufficient to definitively establish its efficacy. Therefore, further research involving a larger cohort over a more extended period is recommended. 

Grebe et al. [105] investigated the impact of intravenous vitamin C administration on endothelial function in patients with OSA. Ten untreated patients with OSA and ten age- and sex-matched control subjects without sleep-disordered breathing were enrolled in the study. Endothelial dysfunction can be an indirect marker of oxidative stress, and it can be assessed noninvasively through an ultrasound measurement of the brachial artery flow-mediated dilation (FMD), a valid indicator of endothelial function. This technique involves evaluating the change in arterial diameter in response to increased shear stress following transient forearm ischemia. The study found a significant enhancement in brachial artery flow in OSA patients following vitamin C administration, an effect not observed in the control group (*p* < 0.01). Vitamin C was selected due to its well-documented antioxidant properties and its beneficial effects on endothelial function in conditions characterized by elevated oxidative stress, such as diabetes mellitus, hypercholesterolemia, hypertension, and heart failure. The underlying mechanism involves a reduction in circulating reactive oxygen species and the restoration of nitric oxide bioavailability, thereby contributing to vascular homeostasis. As with the NAC study, the small sample size is a primary limitation. Furthermore, we believe that FMD may not be the optimal marker for oxidative stress. Future studies should use one of the previously discussed markers to enhance comparability between studies.

Allopurinol, widely prescribed for its urate-lowering effects in conditions such as gout, has also drawn attention for its antioxidant properties: in addition to inhibiting xanthine oxidase, allopurinol has been shown to reduce lipid peroxidation and scavenge free radicals. Although current evidence primarily stems from preclinical models, a study conducted in rats demonstrated a marked reduction in lipid peroxidation products following allopurinol treatment [106]. These findings suggest a potential role for allopurinol in modulating oxidative stress, warranting further investigation in clinical settings involving human subjects.

Notably, we did not find any studies that specifically explore the potential relationship between OSA and acetylsalicylic acid (ASA). Beyond its established antiplatelet effects, ASA has been reported to possess notable antioxidant properties, including free radical scavenging activity [107,108]. ASA has been shown to protect low-density lipoproteins (LDL) from oxidative modification, safeguard vascular tissues from reactive oxygen species, and inhibit protein oxidation through the acetylation of lysine residues or the direct neutralization of hydroxyl radicals. Some of these antioxidant mechanisms are thought to involve the modulation of gene expression, such as the suppression of the transcription factor NF-*κ*B, which plays a pivotal role in inflammatory signaling pathways. Evidence from both in vitro and in vivo studies highlights the multifaceted antioxidant actions of ASA, suggesting that it may exert protective effects at various physiological levels [109]. Given these findings, we believe ASA may represent a promising candidate for antioxidant therapy in OSA, particularly in patients exhibiting the so-called ’aspirin-resistant’ phenotype [110]. However, clinical studies in human populations are required to confirm its efficacy and safety in this context, as this is purely a speculative hypothesis for future research.

This narrative review highlights a significant lack of homogeneity in the literature on oxidative stress biomarkers and, by extension, antioxidant therapies. Although many potential oxidative biomarkers have been proposed, none have yet to achieve clear clinical utility. We believe future studies should aim to identify one or more reliable markers that can be applied consistently in trials of antioxidant therapy to enhance comparability across investigations. Without such standardization, variability in marker selection introduces a bias that undermines a study’s strength.

## 7. Conclusions

Obstructive Sleep Apnea is not solely a disorder of sleep-related breathing, but it also exerts profound systemic effects. The recurring episodes of oxidative stress and intermittent hypoxia associated with OSA contribute to endothelial dysfunction, metabolic dysregulation, systemic inflammation, and an increased risk of cardiovascular complications.

Biomarkers indicative of oxidative stress have shown potential in evaluating disease severity and monitoring individual responses to therapy. However, none of these biomarkers have yet to achieve universal acceptance for routine use in clinical settings. Regarding treatment, CPAP remains the only widely approved intervention. In addition to its primary role in maintaining airway patency, CPAP has demonstrated the ability to reverse several molecular and physiological abnormalities associated with the condition.

Despite CPAP’s effectiveness, residual dysfunctions often persist even after nocturnal oxygenation is normalized. In such cases, pharmacological interventions may play a valuable role. Among these, antioxidant therapies are still in the early stages of development, yet several compounds have shown promising results in preliminary studies and warrant further investigation. It is plausible that, in the future, antioxidants could serve as adjunctive therapies to enhance the management of OSA.

In conclusion, further research is essential to identify novel biomarkers through advanced diagnostic methodologies and to develop new pharmacological treatments. These innovations could offer additional therapeutic benefits beyond those achieved by CPAP alone, ultimately improving patient outcomes in OSA management.

## Figures and Tables

**Table 1:** Effects of Reactive Oxygen Species (ROS) in biological systems. Adapted from Zuo et al. [37]. NLRP3: NLR family pyrin domain containing 3; LTP: Long-Term Potentiation; COPD: Chronic Obstructive Pulmonary Disease; HIF-1*α*: Hypoxia-Inducible Factor-1*α*; IR: Ischemia–Reperfusion; and ALS: Amyotrophic Lateral Sclerosis.

Type	Role	Main Effects
**GOOD**	Cellular activities	Involved in cellular response to stressors, regulates mitochondrial function, expression of certain stress proteins, and antioxidant levels
	Immune system	Activates NLRP3 inflammasomes or other immune-related receptors, helps combat invading pathogens
	Synaptic plasticity	Involved in the formation of LTP
**BAD**	Protein degradation	Leads to protein modification, influences protein translation, and increases the susceptibility of proteins to proteolysis
	DNA damage	Induces mutagenesis, oxidizes nucleotides (guanine is particularly susceptible)
	Muscle damage	Increases fatigue thus reducing muscle function, promotes oxidative damage to muscle protein
**UGLY**	Cancer	Induces DNA mutation, upregulates HIF-1α, which is involved in tumor angiogenesis
	Pulmonary diseases	Enhances inflammation response and damages diaphragm function, contributes to pulmonary diseases such as COPD or asthma
	Cardiovascular diseases	Involved in IR damage, causes hypertension via mechanisms such as lipid peroxidation
	Neurodegenerative diseases	Correlated with neurodegenerative diseases such as Parkinson’s disease, Alzheimer’s disease, and ALS

**Table 2:** Diagnostic cut-off values for oxidative stress biomarkers (OS biomarker) in OSA. N/A, data not available in study; hsCRP, high-sensitivity C-Reactive Protein; TRX, thioredoxin; TBARS, thiobarbituric acid reactive substances; AOPPs, Advanced Oxidation Protein Products; and 8-OHdG, 8-hydroxy-2-deoxyguanosine.

OS Biomarker	Cut-Off	Sensitivity	Specificity	Reference
hsCRP	5.55 mg/L	95%	88%	Suliman et al. [70]
TRX	9.39 ng/mL	91%	78%	Guo et al. [66]
TBARS	N/A	N/A	N/A	Pau et al. [71]
AOPPS	N/A	N/A	N/A	Li et al. [72]
8-OHdG	N/A	N/A	N/A	Chen et al. [73]

**Table 3:** Correlation between oxidative stress biomarkers (OS biomarker) and OSA severity (AHI, ODI, and TSpO_2_ < 90%) reported by Stanek et al. [35]. N/A, data not available in study; hsCRP, high-sensitivity C-Reactive Protein; TRX, thioredoxin; TBARS, thiobarbituric acid reactive substances; AOPPs, Advanced Oxidation Protein Products; 8-OHdG, 8-hydroxy-2-deoxyguanosine; AHI, apnea–hypopnea index; ODI, oxygen de-saturation index; and TSpO_2_ < 90%, percentage of total sleep time with oxygen saturation below 90%.

OS Biomarker	AHI	ODI	TSpO_2_ < 90%
hsCRP	*p* < 0.001	*p* < 0.001	*p* < 0.001
TRX	*p* < 0.05	N/A	N/A
TBARS	*p* < 0.0001	*p* < 0.0001	N/A
AOPPS	*p* < 0.001	*p* < 0.001	*p* < 0.001
8-OHdG	Positive (*p* not specified)	Positive (*p* not specified)	Positive (*p* not specified)

**Table 4:** Main antioxidant agents as therapeutic options.

Agent	Sample	Oxidative Stress Marker	Outcomes	Reference
N-acetylcysteine (NAC)	Human (n = 20)	Lipid peroxidation levels	Reduction in the NAC- treated group compared to the control group (*p* < 0.001)	Sadasivam et al. [104]
Vitamin C	Human (n = 20)	Brachial artery flow-mediated dilation (FMD)	Improved in the Vitamin C-treated group compared to the control group (*p* < 0.01)	Grebe et al. [105]
Allopurinol	Rats	Lipid peroxidation levels	Reduction in the allopurinol-treated group compared to the control group (*p* < 0.05)	Williams et al. [106]

## Data Availability

Due to privacy and ethical considerations, the dataset supporting the reported results can be provided upon request. Interested parties can contact the corresponding author directly via email to request access.
